# Trypsinogen 4 boosts tumor endothelial cells migration through proteolysis of tissue factor pathway inhibitor-2

**DOI:** 10.18632/oncotarget.4949

**Published:** 2015-07-22

**Authors:** Carmen Ghilardi, Antonietta Silini, Sara Figini, Alessia Anastasia, Monica Lupi, Robert Fruscio, Raffaella Giavazzi, MariaRosa Bani

**Affiliations:** ^1^ Laboratory of Biology and Treatment of Metastases, IRCCS-Istituto di Ricerche Farmacologiche Mario Negri, Milan, Italy; ^2^ Laboratory of Cancer Pharmacology, IRCCS-Istituto di Ricerche Farmacologiche Mario Negri, Milan, Italy; ^3^ Clinic of Obstetrics and Gynecology, University of Milan-Bicocca, San Gerardo Hospital, Monza, Italy

**Keywords:** endothelial cells, cancer microenvironment, tumor angiogenesis, serine protease 3 (PRSS3)/trypsinogen 4, tissue factor pathway inhibitor 2 (TFPI-2)

## Abstract

Proteasescontribute to cancer in many ways, including tumor vascularization and metastasis, and their pharmacological inhibition is a potential anticancer strategy.

We report that human endothelial cells (EC) express the trypsinogen 4 isoform of the serine protease 3 (PRSS3), and lack both PRSS2 and PRSS1. Trypsinogen 4 expression was upregulated by the combined action of VEGF-A, FGF-2 and EGF, angiogenic factors representative of the tumor microenvironment. Suppression of trypsinogen 4 expression by siRNA inhibited the angiogenic milieu-induced migration of EC from cancer specimens (tumor-EC), but did not affect EC from normal tissues. We identified tissue factor pathway inhibitor-2 (TFPI-2), a matrix associated inhibitor of cell motility, as the functional target of trypsinogen 4, which cleaved TFPI-2 and removed it from the matrix put down by tumor-EC. Silencing tumor-EC for trypsinogen 4 accumulated TFPI2 in the matrix.

Showing that angiogenic factors stimulate trypsinogen 4 expression, which hydrolyses TFPI-2 favoring a pro-migratory situation, our study suggests a new pathway linking tumor microenvironment signals to endothelial cell migration, which is essential for angiogenesis and blood vessel remodeling. Abolishing trypsinogen 4 functions might be an exploitable strategy as anticancer, particularly anti-vascular, therapy.

## INTRODUCTION

Endothelial cells (EC) line the blood vessels and regulate important physiological and pathological processes such as angiogenesis, metastasis and blood coagulation. Proteases secreted by EC, and in particular perivascular proteases, influence neo-vascularization by activating growth factors and modifying membrane receptors which can induce EC and mural cell migration [1]. Understanding how protease activities regulate EC response to microenvironment signals might help to identify new way to inhibit neo-vascularization in cancer and angiogenesis-related diseases. Among the proteases contributing to cancer are the serine proteases [2]. An association between serine protease 3 (PRSS3) expression and a worseprognosis in cancer patients have been recently shown by microarray data [3–5],but its role in the patho-physiology of tumors is mostly unknown. PRSS3is an atypical member of the trypsinogen family, unaffected by the naturally occurring polypeptide trypsin inhibitors and capable of degrading the Kunitz-type family of serine protease inhibitors [6, 7]. The PRSS3 gene encodes two proteins, mesotrypsinogen and trypsinogen 4 [7]. Mesotrypsinogen is the minor component of the secreted pancreatic trypsinogens (approximately 0.5% of the total proteins in human pancreatic juice). Trypsinogen 4 is “ *extra-pancreatic* ”and is found in the brain, expressed by a subset of neuronal and glial cells (mainly astrocytes). It is also expressed at much lower levels in other tissues and in some human epithelial cell lines [8–11]. To date, the physiological role of trypsinogen 4 is unknown.

We had shown by *in situ* hybridization that the *in vivo* expression of PRSS3 transcript is associated with the tumor vasculature [12]. In the present study, we extend our previous findings and report that trypsinogen 4 is the trypsin family member expressed by the EC, and thata pro-angiogenic environment enhances its expression. It is known that proteases (secreted and membrane-bound) break down extracellular matrix and cellular adhesion molecules, facilitating angiogenesis, invasion, and metastasis [13, 14]. Here we used EC isolated from cancer specimens (tumor-EC) and show thattrypsinogen 4 is required for the migration of tumor-EC promoted by thetumor microenvironment, and exerts its pro-angiogenic action through the inhibition of the tissue factor pathway inhibitor-2 (TFPI-2).

## RESULTS

### Endothelial cells express the trypsinogen 4 isoform of serine protease-3 (PRSS3)

An earlier investigation suggested that human endothelial cells (EC) from umbilical vein (HUVEC) and derma (dMvEC) express serine protease-2 (PRSS2) [15]. Our microarray analyses have suggested that PRSS3 is expressed by EC and from cancer specimens (tumor-EC) [12]. This induced us to isolate tumor-EC (Supplementary Figure 1) to be investigated.

**Figure 1:**
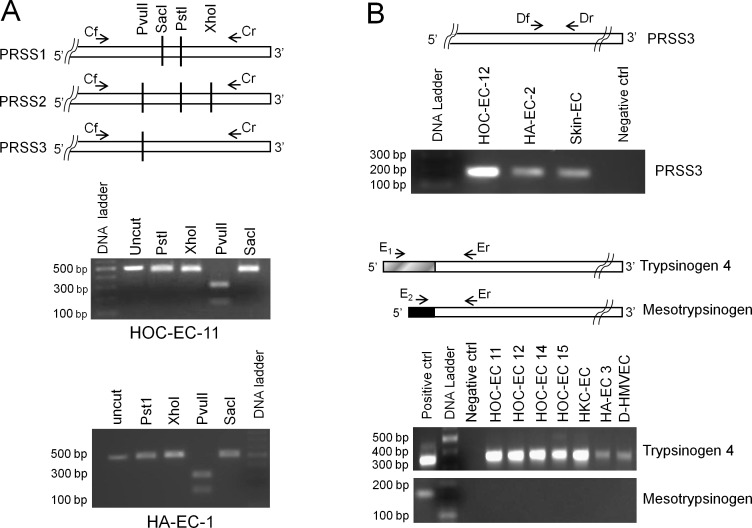
Trypsin expression by endothelial cells RNAof human endothelial cells from ovarian (HOC-EC) and kidney (HKC-EC) carcinomas, and from non-neoplastic tissues such as skin, adrenal gland (HA-EC), derma and lung (D- and L-HMVEC) was reverse-transcribed and PCR-amplified. For the primer sequences see Supplementary Table 1. ***A:*** *Endothelial cells do not express PRSS1 and PRSS2.* Top panel shows the primers (Cf and Cr) common to PRSS1, PRSS2 and PRSS3 used to generate the amplicon, and the restriction sites discriminating the three transcripts. The uncut amplicon and the fragments produced by its digestion with PstI, XhoI, PvuII or SacI enzymes were separated by gel electrophoresis. The lower panels show the results for two representative EC populations, demonstrating that only PvuII can cut, hence indicating that only PRSS3 is expressed. ***B:*** *Endothelial cells express PRSS3, specifically the trypsinogen 4 isoform.* The 5′ terminus alignment of PRSS3, trypsinogen 4 and mesotrypsinogen, is shown with the common (Df, Dr and Er) and the specific (E1 and E2) primers for the different isoforms. The gel electrophoresis pictures show the PCR product of the expected length for PRSS3 (170 bp; top panel) and for trypsinogen 4 (345 bp; middle panel) and the lack of mesotrypsinogen amplification (191 bp; bottom panel) for a number of representative EC populations. (Positive control, HT29 colon cancer cells [10]; negative control, no template).

Restriction enzyme digestion analyses indicated that PRSS3 is expressed by tumor-EC while PRSS1 and PRSS2 are not. As shown in Figure 1A(middle picture, HOC-EC) PvuII was able to cut the amplicon while PstI,SacI and XhoI did not, a result fitting only with the PRSS3 sequence. Accordingly, RT-qPCR using PRSS1 or PRSS2 TaqMan specific assays did notresult in transcript amplification, while the use of PRSS3 assay did (Supplementary Table 2). This finding was confirmed using primers designed to specifically amplify PRSS3, whereby gel electrophoresis revealed amplification of a PCR product of the expected length (Figure 1B, top picture, HOC-EC).

The PRSS3 gene gives rise to either mesotrypsinogen or trypsinogen 4 mRNAs, which share exons 2 through 5 but differ in exon 1. Gel electrophoresis of PCR products obtained usingspecific primers for each isoform, showed that tumor-EC expressed trypsinogen 4 but not mesotrypsinogen (Figure 1B, bottom pictures, HOC-EC and HK-EC).

Similarly, EC from normal tissues, such as skin,adrenal glands (HA-EC), derma and lung (D- and L-HMVEC), expressed PRSS3/trypsinogen 4 but not PRSS1 and PRSS2 (Figure 1A bottom picture and Figure 1B).

Taken together, these results demonstrate that trypsinogen 4 is the trypsin isoform expressed by the human endothelium.

### Tumor-EC over-express trypsinogen4: effect of the microenvironment

To assess the effect of the microenvironment on the expression of trypsinogen 4, we examined EC in two different cultureconditions: either in presence of stimuli mimicking the tumor microenvironment, or in their absence to copy the non-neoplastic setting.

**Figure 2:**
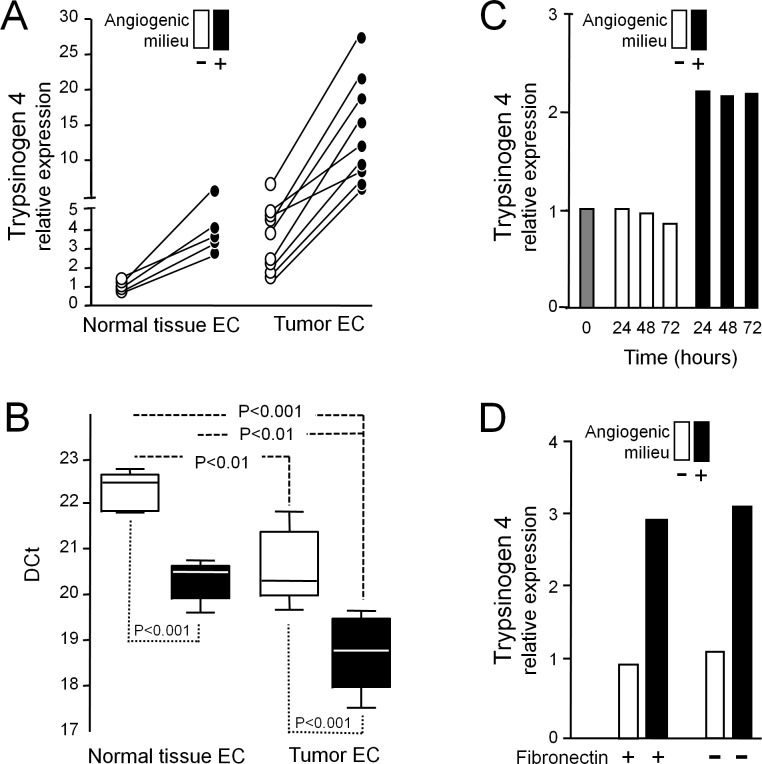
Effect of the angiogenic milieu mimicking a “tumor microenvironment” on trypsinogen 4 expression EC from human cancer and non-neoplastic tissues were exposed (+, black) or not (−, white) to the *in vitro* reconstituted “angiogenic milieu” (i.e. human recombinant VEGF-A, FGF-2and EGF) and trypsinogen 4 expression was assayed by RT-qPCR. ***A:*** *The angiogenic milieu up-regulates trypsinogen 4 expression by EC.* The trypsinogen 4 relativeexpression was quantified for each biological sample according to the comparative DDCt method by arbitrarily assuming as “the reference” calibrator the DCt mean value of the EC from non-neoplastic tissue not exposed to the angiogenic milieu. Shown here are the results of 9 different tumor-EC (isolated from 8 ovarian and 1 kidney carcinoma), and 5 normal tissue-EC (isolated from 3 adrenal gland, 1 skin and 1 lung specimens). ***B:*** *Trypsinogen 4 is expressed more by tumor than non-neoplastic EC.* The box plot shows the DCt values of the tumor-EC (*N* = 9) and the normal tissues-EC (*N* = 5) and the statistical analysis results (one-way ANOVA and Bonferroni Multiple Comparison Test). ***C:*** *Trypsinogen 4 up-regulation is measurable after 24 hours.* Shown here is the expression of trypsinogen 4 byrepresentative ovarian tumor-EC from carcinoma (HOC-EC) embedded or notin the angiogenic milieu for different times. The grey column (time 0) represents the baseline trypsinogen 4 expression (before exposing the cells to the experimental conditions). ***D:*** *The matrix protein fibronectin does not affect trypsinogen 4 expression*. Shown here is the expression of trypsinogen 4 bya representative HOC-EC seeded on either CollagenI or CollagenI plus plasma fibronectin coated tissue culture plastic and exposed (black) or not (white) to the angiogenic milieu.

VEGF-A, FGF-2 and EGF, factors which characterize the angiogenic milieu, were able to significantly increase trypsinogen 4 expression by tumor-EC (Figure 2A and 2B). The up-regulation was measurable after 24h of exposure and was maintained at 72h (Figure 2C), regardless of fibronectin (Figure 2D). Similarly, EC from normal tissues reacted to the angiogenic milieu up-regulating trypsinogen 4, but the amount of the transcript remained significantly lower in comparison to tumor-EC (Figure 2A and 2B); indeed it was similar to that of tumor-EC in absence of the angiogenic milieu.

### A pro-angiogenic environment promotes tumor-EC migration by up-regulating trypsinogen 4

**Figure 3:**
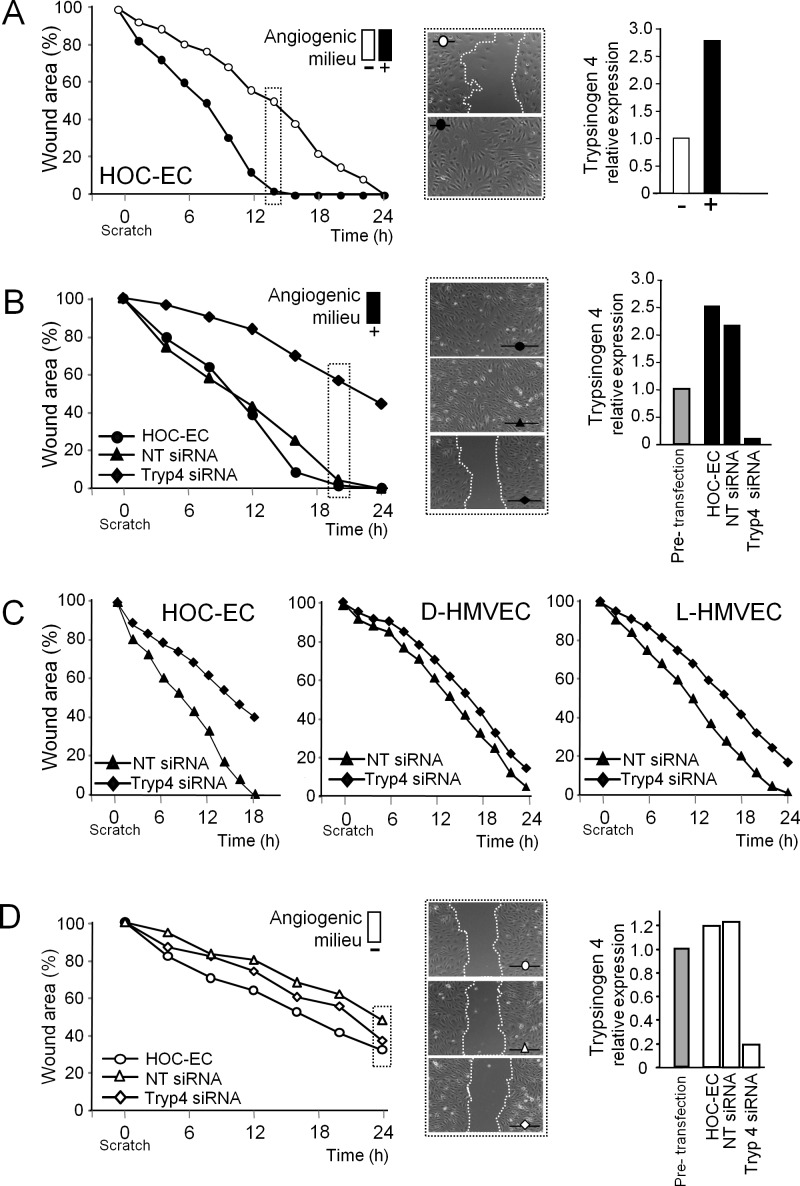
Effect of trypsinogen 4 silencing on the migration ability of tumor endothelial cells Migration was assessed in a wound healing assay; wound closure was monitored by Cell^®^Imaging Station and quantified by measuring the wound area over time and comparing it with the initial size (assumed as 100%). Results are the mean of three replicates. Representative captions of the wounds are shown. Tumor-EC from ovarian carcinoma (HOC-EC) and normal-EC from derma and lung (D- and L-HMVEC) were transfected with either trypsinogen 4 (Tryp4) or not-targeting (NT) siRNA. Silencing was assessed by RT-qPCR 24h later (i.e. at the time of wounding); trypsinogen 4 expression before transfection was arbitrarily taken as the reference. Different populations of HOC-EC were investigated (populations 13, 15, 17 and 18); shown here are representative results. ***A:*** *The angiogenic milieu triggers the migration of tumor-EC.* Shown here are the results of HOC-EC embedded (+, black) or not (−, white) in the *in vitro* reconstituted “angiogenic milieu” (i.e. human recombinant VEGF-A, FGF-2 and EGF). ***B:*** *Trypsinogen 4 silenced tumor-EC are not longer able to close the wound.*Shown here are the results of HOC-EC, silenced (Tryp4 siRNA) or not (NTsiRNA and wild type), assayed in presence of the angiogenic milieu. ***C:*** *Trypsinogen 4 does not have an impact on the migration of EC from normal tissues.* Shownhere are the results of HOC-EC, D-HMVEC and L-HMVEC silenced (Tryp4 siRNA) or not (NT siRNA) assayed in presence of the angiogenic milieu. ***D:*** *Tumor-EC deprived of the angiogenic milieu migrates weakly regardless of trypsinogen 4.* Shownhere are the results of HOC-EC silenced (Tryp4 siRNA) or not (NT siRNA and wild type) assayed in absence of the angiogenic milieu.

Pro-angiogenic cues in the tumor microenvironment can favor tumor-EC migration, a critical step for angiogenesis and vascular remodeling. Accordingly, tumor-EC isolated from ovarian carcinoma (HOC-EC) were able to close the wound much fasterwhen in presence of the angiogenic milieu (Figure 3A). To test whether trypsinogen 4 had a role in the increased migration of tumor-EC, we suppressed its transcription using short-interfering RNA (siRNA). Trypsinogen 4-proficient HOC-EC (both wild type and transfectedwith non silencing (NT) siRNA) closed the wound in 20 hours (Figure 3B),while trypsinogen 4-deficient HOC-EC migrated much more slowly (the wound width decreased by only 50% after 24 hours). In contrast, the delay in wound closure by trypsinogen 4-deficient normal-EC from derma (D-HMVEC) and lung (L-HMVEC) was negligible (Figure 3C). Altogether these results demonstrate that in presence of an angiogenic milieu, the depletion of trypsinogen 4 profoundly impairs the migratory capabilities of tumor-EC, while it does not affect the migration of EC from normal tissues.

In the absence of the angiogenic milieu (Figure 3D),trypsinogen 4-deficient and proficient HOC-EC migrated equally poorly, suggesting that the microenvironment has a profound influence on the level of trypsinogen 4 required to boost tumor-EC migration.

**Figure 4:**
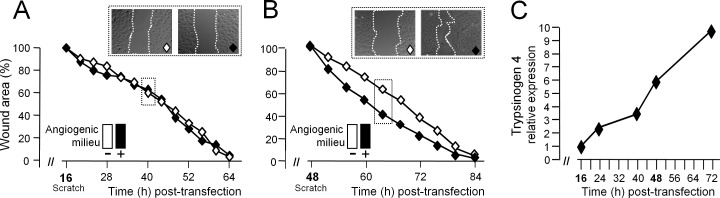
Trypsinogen 4 mediates the microenvironment-induced migration of tumor endothelial cells HOC-EC(population 19) were transfected with trypsinogen 4 specific siRNA and plated in presence (+, black) or absence (−,white) of in the *in vitro* reconstituted “angiogenic milieu” (i.e. human recombinant VEGF-A, FGF-2 and EGF) and assayed at two different time points. ***A*** and ***B:*** *Migration of trypsinogen 4-silenced tumor-EC: effect of the microenvironment.* Wounding was done 16h (**A)** and 48h (**B)** after transfection. Wound closure was monitored by Cell^®^Imaging Station and quantified by measuring the wound area over time and comparing it with the initial size (assumed as 100%). Results are the mean of three replicates with representative captions of the wounds. ***C:*** *Time course of trypsinogen 4 re-expression by silenced tumor-EC in presence of the angiogenic milieu*. The kinetic of trypsinogen 4 re-expression was assessed by RT-qPCR at the times indicated. The minimum expression (i.e. 10% of the not-transfected HOC-EC) was observed 16 hours after transfection and was assumed as the “reference” expression (maximum silencing effect).

To further demonstrate that the angiogenic milieu was able to stimulate the migration of tumor-EC by increasing trypsinogen 4 expression, HOC-EC were transfected with trypsinogen 4 siRNA and seeded either with or without the angiogenic milieu (VEGF-A, FGF-2 and EGF), and their ability to repair the wound was measured at two different time points. The migration of HOC-EC wounded 16 h after transfection, when maximum silencing was achieved (Figure 4C), was unaffected by the angiogenic milieu (Figure 4A). In contrast, the angiogenic milieu enhanced the migration of HOC-EC wounded 48 h after transfection (Figure 4B), the time at which the silencing effect was fading and the expression of trypsinogen 4 was partially restored (Figure 4C).

These findings demonstrate that when the abilityof tumor-EC to up-regulate trypsinogen 4 is impaired, they are no longer capable of migrating in response to the angiogenic stimulus provided by the tumor microenvironment.

### Trypsinogen 4 enhances tumor-EC migration by acting on tissue factor pathway inhibitor (TFPI-2)

To clarify the mechanism by which trypsinogen 4 affects the migratory capacity of tumor-EC, we investigated its relationship with TFPI-2. TFPI-2, a Kunitz-type serine protease inhibitor, is expressed by EC and has anti-angiogenic properties [16]; characteristics that elected TFPI-2 as a possible substrate and effector of trypsinogen 4.

**Figure 5:**
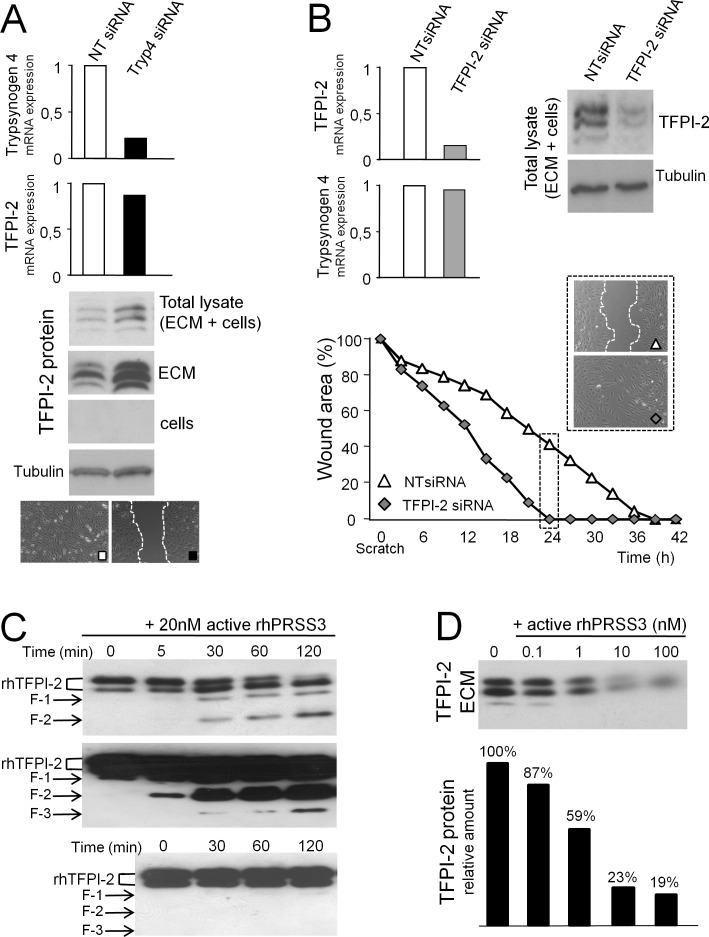
Trypsinogen 4 decreases TFPI-2 availability HOC-EC were transfected with siRNA specific for trypsinogen 4 (Tryp4 siRNA), TFPI-2 (TFPI-2 siRNA) or not-targeting (NT siRNA). Trypsinogen 4 and TFPI-2 mRNAs were quantified by RT-qPCR 24 hours later (expression by NT siRNA HOC-EC was arbitrarily assumed as reference). Three different HOC-EC populations were investigated (populations 23, 25 and 27); shown here are representative results. ***A:*** *Trypsinogen 4-silenced tumor-EC have a much larger amount of TFPI-2 protein.* Protein lysates were prepared 24 hours after transfection. Western blot analysis was done to assess TFPI-2 protein in the total lysate, in the cells and in the extracellular matrix (ECM). Tubulin was used as internal control. Migration was assessed by a wound healing assay. ***B:*** *TFPI-2-silenced tumor-EC close the wound faster.* The wound healing assay was done 24h after transfection and wound closure was monitored by Cell^®^ Imaging Station. TFPI-2 protein was quantified by Western blot analysis. ***C:*** *Active trypsinogen 4 cleaves TFPI-2 in a cell-free system.* Recombinanthuman TFPI-2 (500 nM) was incubated at 37°C with 20 nM of active recombinant human PRSS3 (rhPRSS3). The reaction was stopped at the timesindicated and the TFPI-2 protein was analysed by western blot. The arrows indicate the different TFPI-2 digestion products. Short and long membrane exposure time are shown to better illustrate how intact rhTFPI-2 fades (two bands, top panel) and the appearance of a number of lower molecular weight products (middle panel). The same amount of TFPI-2 was incubated at 37°C without trypsinogen 4 (bottom panel, long exposure). ***D:*** *Active trypsinogen 4 displaces TFPI-2 from the extracellular matrix of tumor-EC.* The extracellular matrix deposited by HOC-EC was incubated for 1h at 37°C with different concentrations of rhPRSS3 and TFPI-2 protein was analyzed by western blot.

We found that trypsinogen 4-deficient HOC-EC had much more TFPI-2 protein than their trypsinogen 4-proficient counterpart (Figure 5A), although this was not reflected by a difference in the amount of transcript (Figure 5A). The protein was found in the total lysate but it was undetectable in the cells, in line with a previous report showing that most of the TFPI-2 was released and remained in the extracellular matrix (ECM) [17]. Accordingly, much more TFPI-2 accumulated in the ECM deposited by the slowly migrating HOC-EC silenced for trypsinogen 4 (Figure 5A). In line with this, TFPI-2-silenced HOC-EC migrated much faster and by 24h they had completely closed the wound, while TFPI-2-proficient HOC-EConly halved the wound width (Figure 5B). These results suggest that trypsinogen 4 promotes tumor-EC migration by displacing TFPI-2 from the matrix.

To substantiate this hypothesis, first we investigated the proteolytic activity of trypsinogen 4 on TFPI-2. Recombinant human TFPI-2 (rhTFPI-2) and active trypsinogen 4 (rhPRSS3) proteins were incubated in a cell-free system and the proteolysis of TFPI-2 was monitored by western blot. In a time course experiment, rhPRSS3 cleaved rhTFPI-2, and a number of proteolytic fragments were visible after 5 minutes (Figure 5C, top and middle panels). No spontaneous cleavage was detected in the reaction containing only rhTFPI-2 (Figure 5C, bottom panel), thus demonstrating the direct proteolytic activity of trypsin 4 on TFPI-2.

Next we determined whether trypsinogen 4 could cleave TFPI-2 in a complex biological sample, such as the matrix of tumor-EC. HOC-EC were allowed to deposit TFPI-2 rich ECM, which was thenincubated for 1 hour with active trypsinogen 4 (rhPRSS3). We observed aconcentration-dependent depletion of intact TFPI-2 from the ECM, starting from rhPRSS3 concentrations as low as 1nM (Figure 5D).

Altogether these results indicated that trypsinogen 4 can reduce the extracellular availability of TFPI-2 to favor the migration of tumor-EC mediated by the tumor microenvironment.

## DISCUSSION

The tumor milieu is a complex and highly dynamicenvironment, providing important signals for tumor development and progression. During tumor angiogenesis and blood vessel remodeling, endothelial cells (EC) form new vasculature in response to environmentalcues. These processes primarily require perivascular proteases, such asproteinases/matrix metalloproteinases produced by the EC [1].

**Figure 6:**
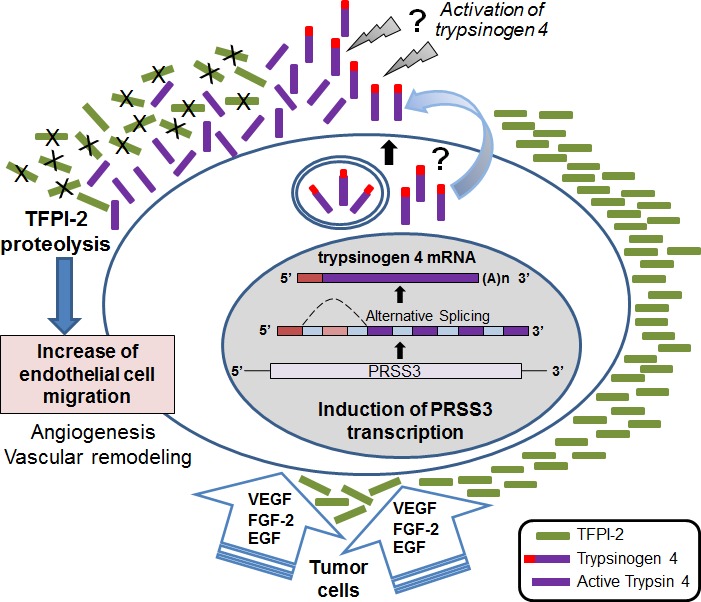
Tumor endothelium manages angiogenesis and blood vessel remodeling through theangiogenic factors-mediated trypsinogen 4 up-regulation and cleavage ofextracellular TFPI-2 The cartoon shows the proposed scenario: the tumor microenvironment (VEGF-A, FGF-2 and EGF provided by the cancer cells and stroma) triggers and sustains the expression of trypsinogen 4 by the endothelium. Upon secretion and activation, trypsinogen 4 cleaves TFPI-2, particularly rich in the endothelial ECM. The displacement of TPFI-2 permits EC migration and hence favors angiogenesis and blood vessel remodeling. The mechanism of release and activation of trypsinogen 4 remains to be elucidated, although there is evidence indicating its activation by cathepsin B [7].

The present study identifies the proteolytic cleavage of extracellular TFPI-2 by trypsinogen 4 as a new pathway regulating the response of tumor endothelial cell to the factors VEGF-A,FGF-2 and EGF (which characterize the angiogenic milieu), and hence tumor angiogenesis (Figure 6). Our results demonstratefor the first time that i) endothelial cells express the trypsinogen 4 isoform of the serine protease PRSS3; ii) trypsinogen 4 cleaves TFPI-2, and iii) displaces it from the extracellular matrix of tumor-EC; iv) theangiogenic milieu dictates the up-regulation of trypsinogen 4 which is essential for the migration of tumor-EC.

Our findings clearly point to PRSS3 as the trypsin family member expressed by the human endothelium. A previous study [15]suggested that PRSS2 was the main type of trypsin expressed by HUVEC and MvEC. However, the PRSS3 sequence was not fully characterized at that time, so PRSS2 could not be distinguished from PRSS3. In light of the new knowledge we are providing, those results are fully compatible with the expression of PRSS3.

The gene encoding PRSS3 gives rise to two main variants, mesotrypsinogen and trypsinogen 4 [8, 18]. We found that the endothelial cells express trypsinogen 4, consistent with the notion that this isoform is expressed by extra-pancreatic tissues [7]. The active forms of mesotrypsinogen and trypsinogen 4 are identical [9, 18],and the variability present in the N-terminal regions supposedly account for differences in tissue specificity, cellular localization, and extracellular transport, although the role and activity of trypsinogen 4 are mostly unknown.

To our knowledge, this is the first report directly implicating trypsinogen 4 in endothelial functions, as one of the proteases mediating signals initiated by the tumor microenvironment. Trypsinogen 4 did not affect proliferation, but its depletion markedly impaired the migration of tumor-EC elicited by a pro-angiogenic environment. In this study we investigated the events that link trypsinogen 4 and endothelial cell migration, demonstrating that trypsinogen 4 decreases the ECM-associated TFPI-2 availability. TFPI-2 is a member of the Kunitz-type family of serine protease inhibitors thatis constitutively synthesized and secreted in the ECM by human endothelial cells [17]. TFPI-2 has been shown to inhibit tumor angiogenesis [19], most likely by directly decreasing EC migration [20]. Our results not only confirm previous finding, but also demonstrate forthe first time that TFPI-2 is a direct substrate of trypsinogen 4 whichmost likely hydrolyses TFPI-2 at the Kunitz-type domains. The proteolytic cleavage leads to the inactivation of TFPI-2, thus blocking its ability to inhibit the migration of tumor-EC.

TFPI-2 has been described as a tumor suppressor gene that can counteract the metastatic potential of tumor cells [21]. Accordingly, its down-regulation in malignant cells, by either histone deacetylation or promoter hypermethylation, has been associated with disease progression [22, 23]. Hence, the proteolytic inactivation of TFPI-2 by trypsinogen 4 might represent an additional “Loss of Function” mechanism favoring cancer by increasing angiogenesis.

Active trypsin 4 has never been detected in cancer, but few reports suggest the higher expression of PRSS3 (i.e. mRNA by microarray) associated with a worse prognosis in NSCL, pancreatic, and prostate cancer [3–5]. Recent studies evaluating the consequences of PRSS3 over-expression or silencing in tumor cells indicated that its activity favors tumor progression [4, 5, 24]. Trypsinogen 4 might therefore have more roles than our current observations suggest and promote cancer through multiple mechanisms, including enhancement of cancer cells invasiveness necessary for metastasis, and endothelial cell migration necessary for neovascularization.

Many proteolytic enzymes, are responsible for reshaping the tumor microenvironment [1]. Their activity is dictated by the formation of molecular and functionalnetworks occurring among proteases, their endogenous inhibitors and substrata, as well as factors regulating their expression and interaction including the crosstalk with regulatory miRNA networks, as recently shown for members of the trypsin-like kallikrein family [25]. Due to this complexity, the contribution of each protease to cancer-related biological processes is difficult to discern. Our findings showing that depletion of trypsinogen 4 is sufficient to impairmigration of tumor endothelial cells suggest a major role for this protease in tumor angiogenesis and blood vessel remodeling. Unfortunately, the lack of trypsinogen 4 orthologues in mice and zebrafish hinders the development of experimental models to investigate its specific function in developmental and pathologic angiogenesis.

We clearly demonstrated that both tumor- and normal tissue- EC respond to the angiogenic milieu by up-regulating trypsinogen 4, suggesting a gene expression regulatory circuitry independent from the pathological or anatomical origin of EC. Even so, trypsinogen 4 expression by tumor-EC is much higher than normal tissue-EC and accordingly, it characterizes the cancer vasculature *in vivo* [12]. EC must dynamically coordinate changes concerning their crosstalk with the surroundings, including when to start and stop moving [26]. Our findings suggest that tumor-EC adapt to the persistent *in vivo* stimulation of the tumor environment by modifying their gene expression and phenotype, and at the same time retain the ability to sense and respond to the changes in the surroundings in a continuous process of vessel formation and remodeling. Blocking the up-regulation of trypsinogen 4 hindered the migration of tumor-EC elicited by the angiogenic milieu, whereas normal tissue-EC remain unaffected. The specificity of this effect is important in view of a selective therapy targeting the cancer vasculature while sparing normal vascular districtsto avoid adverse effects.

The present study brings to light a previously unrecognized pro-angiogenic role for trypsinogen 4 through the inductionof tumor-EC migration, indicating that this protease is a new, potential actionable target for anti-vascular and anticancer treatments.

## MATERIALS AND METHODS

### Isolation, culture and characterization of EC from human tissues

EC were isolated from left-over neoplastic tissues of patients undergoing therapeutic surgery, with their full informed consent. Collection of tissue samples was approved by the Local Scientific Ethical Committee (San Gerardo Hospital, Monza, Italy) in compliance with the Declaration of Helsinki. EC isolation has been previously described in detail [12, 27]. Briefly, tissue specimens were digested by type I collagenase (Sigma-Aldrich) and the suspension was plated onto Collagen I plus fibronectin (BD Biosciences). Six to ten days later, EC were purified using anti-CD31 antibody-coated magnetic beads (Life Technologies) and cultured as described [12]. To confirm their endothelial origin, cell cultures were analyzed for the expression of von Willebrand Factor (vWF), platelet-endothelial celladhesion molecule-1 (CD31/PECAM-1), and alpha-smooth muscle Actin (alpha-SMA) all by immunohistochemistry, and also for the capability to uptake low-density lipoprotein (LDL). The results are reported in Supplementary Figure 1. Dermal and lung human microvascular EC (D-HMVEC and L- HMVEC) were purchased from Clonetics^®^.

For experimental purposes, EC were analyzed in the presence or absence of an “angiogenic milieu” reconstituted *in vitro,* namely human recombinant vascular endothelial growth factor (VEGF-A; 10ng/ml), fibroblast growth factor-2 (FGF-2; 2 ng/ml) and epidermal growth factor (EGF; 10 ng/ml) [12].

### RNA isolation

Total RNA was isolated using Trizol^®^(Life Technologies), potential genomic DNA contamination was removed by DNAse (Ambion) treatment followed by RNA CleanUp with RNeasy Mini Kit (Qiagen) according to the manufacturer’s recommendations. The purity and integrity of the RNA were checked by gel electrophoresis or by Agilent Bioanalyzer 2100 (Agilent Technologies), and the concentration was determined with NanoDrop 1000 spectrophotometer (Thermo Scientific).

### RT-PCR and restriction digestion analysis

Primer sets for PCR amplification were designed with Primer3 software (http://frodo.wi.mit.edu/cgi-bin/primer3/primer3_www.cgi)according to the target sequences (Supplementary Table 1) and synthesized by Sigma-Aldrich. One microgram of total RNA was reverse-transcribed for 50 min at 42°C with SuperScriptTM Reverse Transcriptase (Life Technologies) and the reverse primer. One or two μL were then PCR amplified in a 50 μL reaction mixture containing 200 nM primers, 200 μM dNTPs, 1.25 U AmpliTaq Gold and 1.5mM MgCl_2._The thermal condition was: denaturation at 94°C for 10 min, amplification for 35 cycles (denaturation at 95°C for 30s, annealing at 55°C for 30s, extension at 72°C for 30s), and final extension at 72°C for 10 min. The PCR products were separated by gel electrophoresis in a 1.2% agarose gel.

PCR products were gel-purified with Ultrafree DA(Millipore), concentrated with Microcon YM-50 Device (Millipore), and digested for 2h at 37°C with 10U of either PstI, XhoI, PvuII or SacI (Roche Applied Science). The digested fragments were separated by gel electrophoresis in 1.2% agarose gel.

### Quantitative real-time PCR (RT-qPCR)

One microgram of total RNA was reverse-transcribed using the High Capacity cDNA Reverse Transcription Kit and Random Hexamer primers (Applied Biosystems) according to the manufacturer’s protocols. A control RT reaction was set up for each sample without the reverse transcriptase. Quantitative RT-PCR reactions were done in duplicate on the 7900HT Fast Real-Time PCR System (Applied Biosystems). Specific TaqMan^®^ Gene Expression Assays were purchased from Applied Biosystems (Supplementary Table 2). PRSS1, PRSS2, PRSS3 and TFPI-2 expression was normalized to the human 18s rRNA for each EC population (DCt = Ct _target gene_ - Ct _18s_). The DCt were statistically analysed by one-way ANOVA followed by Bonferroni Multiple Comparison Test to compare EC isolated from neoplastic and normal tissues. Fold differences were calculated by the comparative DDCt method.

### Knockdown of trypsinogen 4 and TFPI-2 using small interfering RNA (siRNA)

Trypsinogen 4 and TFPI-2 knockdown was achieved using small interfering RNA. Predesigned siRNA for trypsinogen 4 (Si00693791 and Si00693805), TFPI-2 (GS7980) and negative -non silencing- control siRNA (1022076) were obtained from Qiagen and transfected into EC. Briefly, the cells were detached with StemPro^®^ Accutase^®^(Life Technologies), gently mixed with the nucleic acid (1.1×106 cells in 800 μl medium containing 10% FBS and 200 nM siRNA), and transferred into the electroporation 4mm gap cuvette (VWR International). Electroporation was performed with the following setting: one pulse, pulse length 30 ms, voltage 200V using the Gene Pulser^®^apparatus and capacitance (Biorad). Cells were plated and used for subsequent analyses. The knockdown efficiency was assessed by RT-qPCR.

### Wound-healing assays

Endothelial cells migration was assayed by the wound healing assay [28],that is particularly suitable for studies on the effects of EC interaction with extracellular matrix (ECM). Briefly, EC (2.5×104 cells/insert well) were seeded on fibronectin in Culture-Inserts (Ibidi)and let to form a monolayer before removing the culture-insert to obtain a cell-free gap of 500 μm. Each well was rinsed with phosphate-buffered saline (PBS) and fresh medium (with or without the angiogenic milieu as indicated) was added. The closure of the gap was monitored by Cell^R - Imaging Station (Olympus) and pictures were acquired every 30 min for 24-48h. Migration was quantified using Image Jsoftware (imagej.nih.gov/ij/).

### TFPI-2 hydrolysis

The direct hydrolysis of TFPI-2 by PRSS3 was monitored in a time course experiment, incubating 500 nM recombinant human TFPI-2 (rhTFPI-2, R&D Systems) with 20 nM active recombinant human PRSS3 (rhPRSS3, R&D Systems) at 37°C in assay buffer (50mM Tris, 0.15M NaCl, 10mM CaCl2, 0.05% Brj-35, pH 7.5), following the manufacturer’s recommendations. Samples were drawn at intervals, mixed with Laemmli sample buffer, denatured and stored at −20°C until Western blot analysis.

The PRSS3 depletion/displacement of TFPI-2 from the extracellular matrix (ECM) deposited by EC was also checked. The ECMwas obtained according to a previously established protocol [29]. Briefly, the monolayer of EC was washed three times with PBS, then destroyed by 3 minutes incubation with 0.5% Triton X-100, 4mM NH_4_OH inPBS at room temperature. Wells were repeatedly washed with PBS to remove cell debris, and the remaining ECM was then washed twice with assay buffer. Different concentrations (0.1 nM to 100nM) of active rhPRSS3 diluted in assay buffer, were added and incubated for 1h at 37°C. The ECM was collected in Laemmli sample buffer, denatured and stored at −20°C until Western blot analysis. For total lysates (ECM pluscells), the EC monolayer was lysed in 50mM TrisHCl pH8, 10mM EDTA, 1% Triton X-100, 0.02% NaN_3_, and Protease Inhibitor Cocktail (Roche Applied Science) on ice.

### Western blot analysis

Protein concentrations were measured using the Bio-Rad Bradford protein assay. The proteins were separated by 12% SDS-polyacrylamide gel electrophoresis, transferred to IMMOBILON PVDF membranes (Millipore) and incubated overnight at 4°C in blocking buffer containing 2% ECL blocking reagent (GE Healthcare) and 0.1% Tween 20 in PBS. The membranes were probed with either anti TFPI-2 (1:500, SantaCruzBiotechnology) or anti α-tubulin (1:1000, Sigma-Aldrich) antibody for 1h at room temperature. Antibody binding was detected with anti-mouse IgG-HRP (1:10,000, Sigma Aldrich) for 45min at room temperature. The signals were detected with ECL Prime (GE-Healthcare). Densitometry of protein bands was analysed with GelPro Analyzer Software (Media Cybernetics).

## SUPPLEMENTARY MATERIAL FIGURES AND TABLES


