# Supplementary material for: Trypsinogen 4 boosts tumor endothelial cells migration through proteolysis of tissue factor pathway inhibitor-2
Source: Oncotarget. 2015 Jul 22;6(29):28389–400. doi: 10.18632/oncotarget.4949 (PMC4695067; doi:10.18632/oncotarget.4949)
Supplement: Supplementary file 1 [file oncotarget-06-28389-s001.pdf]

# Trypsinogen 4 boosts tumor endothelial cells migration through proteolysis of tissue factor pathway inhibitor-2

**Supplementary Material**

| Cord formation | LDL uptake | CD31 | vWF | aSMA |
|---|---|---|---|---|
| | % positive cells 99.2 (96.9-100) | % positive cells 99.2 (96.5-100) | % positive cells 99.9 (99.6-100) | % positive cells 0.7 (0-1.4) |

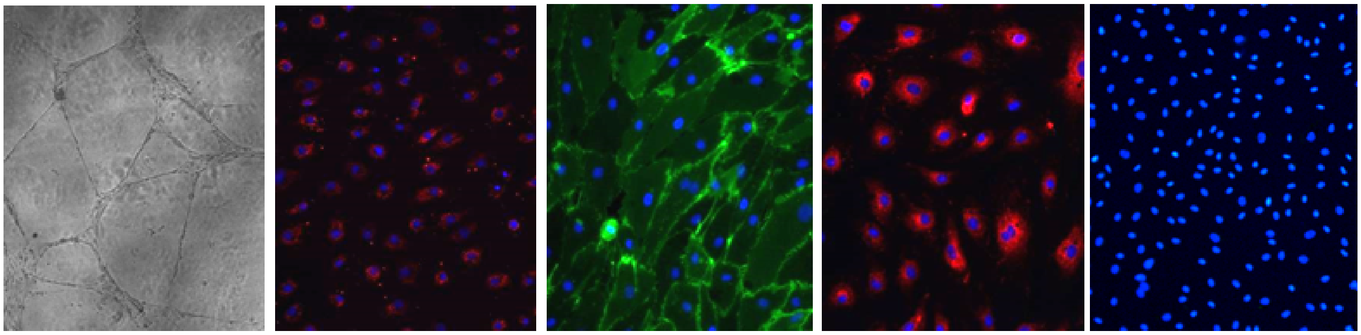

**Supplementary Figure 1: Characterization of endothelial cells**

Endothelial cells isolated from human tumor tissues were characterized to confirm their endothelial origin. Cells formed cord-like structures on matrigel and took up LDL (red), stained positively for the typical endothelial markers CD31 (green) and von Willebrand Factor (red), but not for αSMA, typical of smooth muscle cells (blue, DAPI-counterstained nuclei). Percentages of positive cells were calculated by analyzing the immunofluorescence results for not less than 500 cells. The values [average (range)] for 17 different endothelial cells populations are shown, together with representative captions.

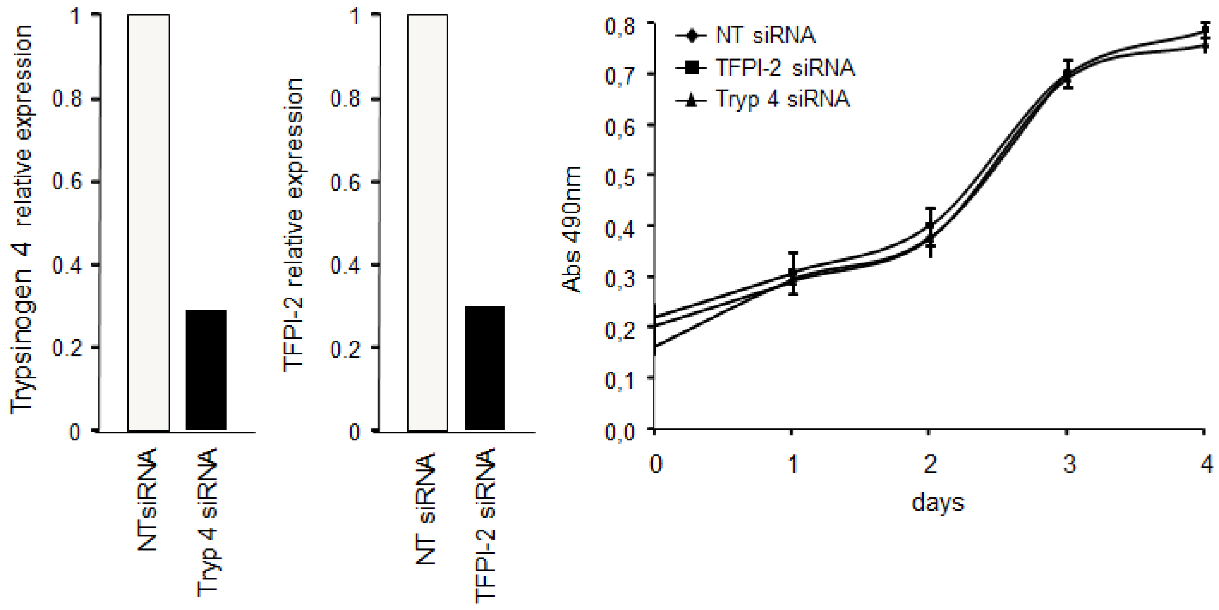


**Supplementary Figure 2: Effect of trypsinogen 4 and TFPI-2 silencing on tumor EC proliferation**

The proliferative activity of endothelial cells transfected with trypsinogen 4, TFPI-2, or not targeting siRNA was the same. Proliferation was measured using the CellTiter96® AQueous Non-Radioactive Cell Proliferation Assay (Promega). Briefly, $3x10^3$ HOC-EC were plated into 96-well plates (six replicates) and placed at 37°C in 5% $CO_2$. After 3h (time=0), 1, 2, 3 and 4 days 20 µl MTS solution were added to each well and incubated for 3h at 37°C in 5% $CO_2$. The plates were thoroughly shaken, and the absorbance at 490nm was recorded with a microplate reader (Infinite®200, Tecan).

**Supplementary Table 1. Primers for RT-PCR analysis**

The primer sequences used for RT-PCR with the amplicon lengths and target transcripts.

| Primer pair | Target transcript | Primer | Primer Sequences | Amplicon length |
|---|---|---|---|---|
| C | PRSS1 (NM_002769)<br>PRSS2 (NM_002770)<br>PRSS3 (NM_007343, NM_002771) | Forward<br>Reverse | 5' CAGGTGAGACTGGGAGAGCACAA 3'<br>5' ATAGTTGTAGACCTTGGTGTAGACTCCAGG 3' | 498 bp |
| D | PRSS3<br>(NM_007343, NM_002771 ) | Forward<br>Reverse | 5' CCTAAATACAACAGGGACACTCTGGAC 3'<br>5' GTCAGCACCAAAGCTCAGAGTGTTG 3' | 170 bp |
| $E_1$+Er | TRYPSINOGEN 4<br>(NM_007343) | Forward<br>Reverse | 5' ATGTGCGGACCTGACGAC 3'<br>5' GCAGTGAGCTGCTGATACCAC 3' | 345 bp |
| $E_2$+Er | MESOTRYPSINOGEN<br>(NM_002771) | Forward<br>Reverse | 5' ATGAATCCATTCCTGATCCTTGC 3'<br>5' GCAGTGAGCTGCTGATACCAC 3' | 191 bp |

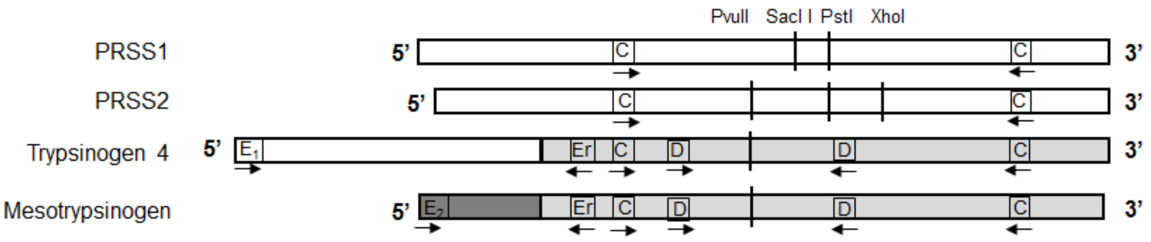

**Supplementary Table 2. Gene expression assays for quantitative RealTime PCR**

Shown are the Specific TaqMan® Gene Expression Assays used for RT-qPCR: the last column indicates whether the amplification was successful (+) or not (-) when RNA from tumor-EC was assayed (N= 7)

| Target transcript | Assay ID | Product length | Tumor EC |
|---|---|---|---|
| PRSS1 (NM_002769) | Hs00605631_g1 | 108 bp | - |
| PRSS2 (NM_002770) | Hs00828418_gH | 150 bp | - |
| PRSS3 (NM_007343, NM_002771) | Hs00605637_m1 | 157 bp | + |
| TFPI-2 (NM_006528) | Hs00197918_m1 | 84 bp | + |
| 18s rRNA | Hs99999901_s1 | 187 bp | + |
